# Targeting DAMPs by Aspirin Inhibits Head and Neck Cancer Stem Cells and Stimulates Radio-Sensitization to Proton Therapy

**DOI:** 10.3390/cancers17132157

**Published:** 2025-06-26

**Authors:** Tea Vasiljevic, Emilija Zapletal, Marko Tarle, Iva Bozicevic Mihalic, Sabrina Gouasmia, Georgios Provatas, Kristina Vukovic Djerfi, Danko Müller, Koraljka Hat, Ivica Luksic, Tanja Matijevic Glavan

**Affiliations:** 1 Laboratory for Personalized Medicine, Division of Molecular Medicine, Rudjer Boskovic Institute, Bijenicka 54, 10000 Zagreb, Croatia; tvasilj@irb.hr (T.V.); emilija.zapletal@irb.hr (E.Z.); kristina.vukovic.djerfi@irb.hr (K.V.D.); 2 Department of Maxillofacial Surgery, Dubrava University Hospital, Gojko Šušak Avenue 6, 10000 Zagreb, Croatia; tarlemarko1@gmail.com (M.T.); koraljkahat@gmail.com (K.H.); luksic@kbd.hr (I.L.); 3 School of Dental Medicine, University of Zagreb, 10000 Zagreb, Croatia; 4 Laboratory for Ion Beam Interactions, Division of Experimental Physics, Rudjer Boskovic Institute, Bijenicka 54, 10000 Zagreb, Croatia; iva.bozicevic.mihalic@irb.hr (I.B.M.); sabrina.gouasmia@irb.hr (S.G.); georgios.provatas@irb.hr (G.P.); 5 Department of Pathology and Cytology, Dubrava University Hospital, Avenija Gojka Šuška 6, 10000 Zagreb, Croatia; danko.mueller@yahoo.com; 6 School of Medicine, University of Zagreb, Šalata 2, 10000 Zagreb, Croatia

**Keywords:** cancer stem cells Toll-like receptor 3 (TLR3), damage-associated molecular patterns (DAMPs), migration, aspirin, γ-irradiation, proton irradiation

## Abstract

Background: Cancer stem cells (CSCs) are a subpopulation of cancer cells known for their self-renewal capacity, tumorigenicity, and resistance to treatment. Toll-like receptor 3 (TLR3) plays a complex role in cancer, exhibiting both pro-apoptotic and pro-tumorigenic effects. This study investigates the pro-tumorigenic role of TLR3, specifically its impact on CSCs in head and neck cancer. Methods: We have investigated Detroit 562, FaDu and SQ20B cell lines, the latter being stably transfected with a plasmid containing inducible shRNA for TLR3, by cultivating them to form tumor spheres in order to study CSCs. Results: Our findings demonstrate that TLR3 activation promotes stemness in head and neck cancer cell lines. This is evidenced by increased tumor sphere formation, promotion of epithelial-to-mesenchymal transition (EMT), upregulated stemness gene expression, and elevated aldehyde dehydrogenase (ALDH) activity. Conditional TLR3 knockdown abolished tumor sphere formation, confirming its important role. Furthermore, TLR3 activation triggers the secretion of damage-associated molecular patterns (DAMPs) into the tumor microenvironment, leading to increased cancer cell migration. This was inhibited by DAMP inhibitors. In patient tissue samples, we observed co-localization of TLR3 with stemness markers CD133 and ALDH1, as well as with heat shock protein 70 (HSP70) and receptor for advanced glycation end products (RAGE). We then explored potential CSC-targeted therapies, initially combining the apoptosis inducer poly (I:C) with DAMP inhibitors and γ-irradiation. While this combination proved effective in adherent cells, it failed to eliminate tumor spheres. Nevertheless, we discovered that proton radiotherapy, particularly when combined with aspirin (HMGB1 inhibitor) and poly (I:C), effectively eliminates CSCs. Conclusions: This novel combination holds promise for the development of new therapeutic strategies for head and neck cancers, particularly given the promising results of proton therapy in treating this disease.

## Simple Summary

This article investigates the pro-tumorigenic role of Toll-like receptor 3 (TLR3) and its influence on cancer stem cells (CSCs) in head and neck cancer. We demonstrate that TLR3 activation promotes stemness in head and neck cancer cell lines. Furthermore, it triggers the secretion of damage-associated molecular patterns (DAMPs), increasing cancer cell migration—an effect that is reversible with common drugs serving as DAMP inhibitors like aspirin and metformin. Importantly, this study identifies a potential therapeutic strategy targeting CSCs, which are often resistant to conventional radio- and chemotherapy, using a combination of aspirin, poly (I:C), and proton irradiation.

## 1. Introduction

Head and neck cancer, the sixth most prevalent cancer globally, presents a significant clinical challenge due to its high morbidity, poor survival rates, and limited therapeutic options such as surgery, conventional chemotherapy, and irradiation. The frequent occurrence of relapse may be attributed to cancer stem cells (CSCs), a distinct population of tumor cells capable of self-renewal, differentiation, and evasion of both radio- and chemotherapy. A recent study established the importance of CSCs for head and neck squamous cell carcinoma (HNSCC) malignant progression by revealing that a CSC^high^(ALDH1^high^p75NTR^high^)/E-cadherin^low^ (epithelial marker) immunohistochemistry panel at the invasive tumor front predicts poor prognosis in oral squamous cell carcinoma [1]. Another recent study revealed that p16-status, higher expression of the CSC markers (SLC3A2 and CD44), and larger tumor volume were associated with increased risk of high-dose failure after primary radiotherapy in HNSCC [2]. A recent analysis of laryngeal squamous cell carcinoma by single-cell RNA sequencing uncovered that this type of CSC exhibits high expression of stem cell marker genes such as CD133, ALDH1A1, and SOX4, and increased activity of tumor-related signaling pathways such as hypoxia, Wnt/β-catenin, and Notch [3].

Toll-like receptors (TLRs) are pattern recognition receptors that play a critical role in innate immunity by detecting molecular motifs associated with pathogens. TLR3, localized primarily in endosomes, is specialized in recognizing double-stranded RNA (dsRNA) derived from viral infections. While TLR3 activation can induce apoptosis in some cancer cells [4], research has unveiled a more intricate role in tumorigenesis. In cancer, TLR3 signaling can activate pro-survival pathways, reprogram cellular metabolism, and contribute to the establishment of a supportive microenvironment for metastasis, thereby fostering malignant progression [5,6,7,8,9]. We have recently shown that TLR3 can also be induced by endogenous ligands (DAMPs) derived from HNSCC necrotic cells [10]. In the present study, we further explore this topic and study whether TLR3 activation can increase the expression and excretion of DAMPs from tumor cells, which can bind and induce other TLRs or similar receptors such as RAGE. DAMPs are endogenous molecules that are released in the extracellular space in response to cellular stress or damage from a trauma or a pathogen. DAMPs can be proteins (HMGB1, HSPs, S100 proteins, etc.), lipids or carbohydrates (low molecular weight hyaluronan, serum amyloid A (SAA)), metabolites (ATP, uric acid), and nucleic acids (DNA, RNA, and mtDNA). Once released, they can induce chronic inflammation and initiation of cancer, as well as its progression through increased angiogenesis, EMT, and stemness stimulation or modify the immune response [11].

HNSCC remains a highly heterogeneous disease, made up of different subtypes, primarily HPV-positive and HPV-negative tumors, each with its own unique molecular features and ways of responding to treatment [12]. The rise of immunotherapy, especially immune checkpoint inhibitors like nivolumab and pembrolizumab that target the PD-1/PD-L1 pathway, has opened new treatment possibilities for patients with recurrent or metastatic HNSCC. However, these therapies benefit only a portion of patients [13,14,15]. At the same time, ongoing research into targeted therapies aimed at specific molecular changes, such as EGFR overexpression and mutations in the PI3K pathway, is showing encouraging signs for more personalized treatment options in the future [16]. Improvements in imaging technologies, including robotic systems, endoscopic tools, and image-guided navigation, with advances in surgical techniques, have made tumor removal more precise and helped preserve important functions [17,18]. All these advances in molecular profiling, targeted drugs, and combined treatment approaches are vital to improving outcomes for HNSCC patients, particularly by tackling the challenges of therapy resistance and cancer recurrence.

We aim to investigate the role of TLR3 in the production and secretion of DAMPs, and how these DAMPs influence cancer stem cells (CSCs). Additionally, we examine the potential of combining pharmacological DAMP inhibitors with two different types of radiation—γ-ray and proton—to induce cell death and eliminate CSCs.

## 2. Materials and Methods

### 2.1. Cells and Reagents

Human head and neck cancer cell lines (FaDu and Detroit 562) were maintained in Dulbecco’s modified Eagle medium (Sigma-Aldrich, Schnelldorf, Germany) supplemented with 2 mM L-glutamine and 10% fetal calf serum in a humidified chamber at 37 °C in 5% CO_2_. FaDu and Detroit 562 cell lines were obtained from ATCC. Cell counts and viabilities were determined by Neubauer counting chamber and Trypan Blue exclusion. Poly (I:C) and poly (A:U) were obtained from InvivoGen (San Diego, CA, USA). DAPI was obtained from (Sigma-Aldrich, Schnelldorf, Germany).

Subclones of the SQ20B cell line were established by transfection with a plasmid carrying shRNA directed against TLR3 and inducible by doxycycline (TET-on system), thus allowing conditional knockdown of TLR3 as described previously [6].

### 2.2. RNA Isolation and Real Time PCR

Total RNA was extracted from Detroit 562 cells using the GenElute^TM^ Mammalian Total RNA Miniprep Kit (Sigma-Aldrich, Schnelldorf, Germany) according to the manufacturer’s instructions. RNA concentration and purity were assessed by spectrophotometry (A260/280), and only samples with ratios between 1.9 and 2.2 were used. For each reaction, 0.3 µg of total RNA was reverse-transcribed using the High-Capacity cDNA Reverse Transcription Kit (Applied Biosystems, Branchburg, NJ, USA) with random primers, following the manufacturer’s protocol. Quantitative PCR was performed using SYBR^®^ Green PCR Master Mix (Applied Biosystems, Warrington, UK) on a QuantStudio^TM^ 3 Real-Time PCR System (Applied Biosystems, USA). The amplification protocol included initial denaturation at 95 °C for 10 min, followed by 40 cycles of 95 °C for 30 s, 62 °C for 30 s, and 60 °C for 30 s. Melting curve analysis was performed to confirm the specificity of amplification. Expression levels were normalized to the 28S ribosomal RNA gene as a reference. Relative expression was calculated using the 2^–ΔΔCt method, after confirming comparable amplification efficiencies among target and reference genes. Experimental procedures and reporting follow the Minimum Information for Publication of Quantitative Real-Time PCR Experiments (MIQE) guidelines. Primer sequences are shown in Supplementary Data (Table S1).

### 2.3. Western Blot Analysis

After protein isolation by RIPA buffer and sonication, they were transferred onto a 0.2 µm nitrocellulose membrane. The membranes were blocked with 5% BSA and were stained with primary antibodies from Santa Cruz Biotechnology (Dallas, TX, USA) (1:1000): S100A9, RAGE, HSP70, HMGB1, TLR4, ALDH1, and ABCG2. The membranes were then stained with peroxidase-conjugated secondary antibody (NA934V, Amersham, in a concentration 1:3000) or with Anti-rabbit IgG, HRP-linked Antibody (#7074, Cell Signaling Technology, Danvers, MA, USA) and visualized with the chemiluminescent system (Perkin Elmer, Waltham, MA, USA) and by using Alliance Q9 Mini Chemiluminescence Imaging system (Uvitec, Cambridge, UK). The complete list of antibodies in concordance with MDAR is presented in Supplementary Data (Tables S2 and S3).

### 2.4. ELISA

To determine whether tumor spheres release endogenous ligands (DAMPs) into the microenvironment, we cultured cells in the form of spheres for 3 days and treated them for 24 h with pIC and pAU. S100A8/9 levels in the medium of spheres were measured using S100A8/A9-Human Calprotectin ELISA kit (Abcam, Cambridge, UK, ab267628), and HMGB1 levels in the medium were measured using Human HMGB1/HMG-1 ELISA Kit (Novus Biologicals, Littleton, CO, USA, NBP2-62766) according to manufacturers’ instructions.

### 2.5. ALDH Activity

ALDH activity was analyzed by the kit ALDH Activity Assay Kit (Fluorometric) (Abcam, Cambridge, UK, ab155894) according to the manufacturer’s instructions. As the expression of the enzyme ALDHA1 was the highest in the 2nd generation of spheres, we seeded the cells in low-adherence flasks and treated the spheres from the 2nd generation with pIC/pAU for 24 h. They were then counted, and the same number of cells for all 4 conditions (adherent, control spheres, spheres treated with pIC, and spheres treated with pAU) were lysed according to the instructions from the kit. Enzyme activity after preparation of the reaction mixture was measured by reading on the Infinite^®^ 200 PRO microplate reader (Tecan) with excitation and emission Ex/Em 535/587 nm.

### 2.6. Immunocytochemistry

Adherent Detroit 562 cells were seeded in an 8-well chamber (Nunc^®^ Lab-Tek^®^ Chamber Slide^TM^ system, Thermo Fischer Scientific, Waltham, MA, USA) at a density of 5 × 104 and grown overnight at 37  °C under 5% CO_2_. Immunostaining with primary antibodies for CD133 (1:100, 17A6.1, Merck, Darmstadt, Germany), RAGE, and HSP70 was performed as described previously [19]. Tumor sphere immunofluorescence was performed as described previously [19].

### 2.7. Proliferation Assays

Proliferation of adherent cells was measured by MTT Cell Proliferation Assay. The absorbance was measured at 570 nm using a microplate reader. Cells were plated at 1 × 104 cells/well in a 96-well microtiter plate and treated with different drugs. Each test point was performed in quadruplicate in three individual experiments (biological replicates).

Proliferation of tumor spheres was measured by ViaLight^TM^ Plus Cell Proliferation and Cytotoxicity BioAssay Kit (Lonza, Basel, Switzerland) according to the manufacturers’ instructions. The viability of tumor spheres was assessed by measuring ATP levels in the cells. However, the protocol was slightly changed, and the lysis step was increased to 60 min to ensure complete tumor spheres lysis. The luminescence was measured by a Tecan Spark microplate reader (Tecan, Männedorf, Switzerland).

### 2.8. Clinical Specimens and Immunohistochemistry

Samples were obtained from 7 patients referred to the Clinical Hospital Dubrava (Zagreb, Croatia). All patients had HNSCC. All the clinical samples were obtained and processed according to the guidelines of the Clinical Hospital Dubrava institutional review board, and all patients gave their informed consent to this study (Table 1). The study was conducted in accordance with the Declaration of Helsinki and approved by the Ethics Committee of Clinical Hospital Dubrava, Zagreb, Croatia (2020/2602-02, 26.02.2020) for studies involving human paraffin samples. The biopsies were fixed in formaldehyde and paraffin-embedded. In this work, 2 μm sections were created from paraffin blocks, followed by deparaffinization in a thermostat. After deparaffinization, pre-digestion and staining were conducted in the Ventana BenchMark Ultra apparatus (Roche Diagnostics, Basel, Switzerland) with thermal stands and ULTRA Cell Conditioning Solution. Immunohistochemical staining was performed by an indirect method (DAB staining method) using an automated immunohistochemical system. The optiViewUniversal DAB detection kit (Ventana Medical Systems, Tucson, AZ, USA) was used for visualization, and the incubation with the antibodies lasted 40 min at a temperature of 37 °C. The resulting complex was visualized using hydrogen peroxide and DAB chromogen, which creates a brown precipitate visible under a light microscope. Then, it was stained with hematoxylin for 1 min and passed through an ascending series of alcohol (70–100%) and xylol. The following antibodies were used at a dilution of 1:100 (TLR3 (Abcam, ab62566), RAGE (Santa Cruz Biotechnology, sc-365154), ALDH1A1 (Santa Cruz Biotechnology, sc-374076), HSP70 (Santa Cruz Biotechnology, sc-24), and CD133 (Merck, MAB4399-I). The secondary antibodies used were Goat anti-Rabbit Alexa Fluor 555 and Goat anti-Mouse Alexa Fluor 647 (Invitrogen, Waltham, MA, USA).

### 2.9. Fluorescent Immunohistochemistry

Immunohistochemistry was performed on slides of 2 µm-thick histology sections obtained from paraffin-embedded HNSCC tumor tissue. The slides were deparaffinized in xylene and rehydrated through a descending gradient of ethanol to distilled water. Antigen retrieval was performed by heating the slides in a sodium citrate solution (pH 6.0) at 95 °C for 3 min and then at 85 °C for 10 min and followed by washing and permeabilization in 0.3% PBS with Triton X-100 (PBST) for 20 min. After blocking nonspecific binding with 5% BSA solution for 60 min at room temperature, sections were incubated overnight at 4 °C with primary antibody. The slides were then washed with 0.025% PBST and incubated with the appropriate secondary antibody for 60 min at room temperature. Washing with PBST was followed by washing in a copper sulfate solution for 10 min to quench the autofluorescence. Slides were mounted using the Fluorescence Mounting Medium (DAKO) and analyzed by an inverted confocal microscope, Leica SP8X.

Antibodies were used at a dilution of 1:100 (TLR3 (Abcam, ab62566), RAGE (Santa Cruz Biotechnology, sc-365154), ALDH1A1 (Santa Cruz Biotechnology, Dallas, TX, USA, sc-374076), HSP70 (Santa Cruz Biotechnology, sc-24), CD133 (Merck, MAB4399-I, Darmstadt, Germany). The secondary antibodies used were Goat anti-Rabbit IgG (H+L) Cross-Adsorbed Secondary Antibody, Alexa Fluor 555 and Goat anti-Mouse IgG (H+L) Cross-Adsorbed Secondary Antibody, Alexa Fluorine 647 (Invitrogen, Waltham, MA, USA).

### 2.10. Migration Assay

For migration measurement, cells were plated into a RadiusTM 96-Well Cell Migration Assay plate (Cell Biolabs, Inc., San Diego, CA, USA). Each plate well contains a 0.68 mm circular non-toxic, biocompatible hydrogel Gel Spot to which cells do not adhere. Before the experiment, cells were confluent and quiescent. At the beginning of the experiment, the Gel Spot was removed, and cells then migrated to the empty circular space. Cells were treated with conditioned medium from tumor spheres grown for 4 days and/or treated with different inhibitors of DAMPs for 48 h. Digital images of the gap closure were taken with a digital camera (DinoEye, 1.3 × 5 megapixel resolution) attached to a microscope. Images were analyzed with ImageJ (ImageJ 1.53k).

### 2.11. Proton Irradiation

Proton irradiation of cells was performed at ambient conditions utilizing the Dual Microprobe (DuMi) end station at the Ruđer Bošković Institute Tandem Accelerator facility (Department of Experimental Physics, Laboratory for Ion Beam Interactions) [20]. Samples were irradiated with a 700 keV proton beam, shaped into a 1 cm diameter circular beam, resulting in a 2 Gy dose per irradiation site. Cells were grown on collagen-coated Mylar films and positioned within in-house fabricated Teflon chambers (Figures S1–S3).

### 2.12. Statistics

All experiments have been performed in at least three biological triplicates. Statistical significance was assessed using a two-tailed Student’s t-test, and the results are given as the mean ± SD. *p*-values < 0.05 were considered statistically significant.

## 3. Results

### 3.1. TLR3 Activation Increases the Stemness of HNSCC Tumor Spheres

**Figure 1:**
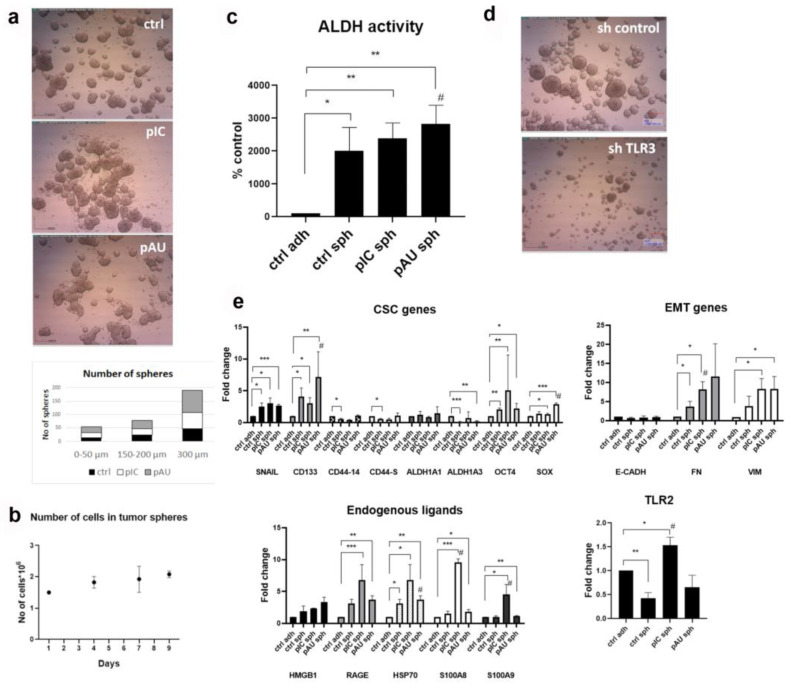
TLR3 activation induces the stemness properties of tumor spheres. (**a**) Poly (A:U) stimulation of Detroit 562 cells stimulates the formation of larger tumor spheres; ctrl—untreated spheres, pIC—tumor spheres treated with poly (I:C), pAU—tumor spheres treated with poly (A:U). At least 5 visible fields were counted. (**b**) The number of cells inside the tumor spheres increases over time, suggesting it is not a result of merging the cells. (**c**) ALDH activity increases in tumor spheres compared to the adherent cells after the stimulation of TLR3; ctrl adh—untreated adherent cells, ctrl sph—untreated spheres, pIC sph—poly (I:C)-treated spheres, pAU sph—poly (A:U)-treated spheres. * *p* < 0.05, ** *p* < 0.02 (compared to the untreated adherent cells), # *p* < 0.05 (compared to the untreated spheres). (**d**) The tumor spheres are dissociated when TLR3 is conditionally knocked down in SQ20B cells transfected with control plasmid (sh control) or plasmid that allows the TLR3 conditional knockdown (shTLR3 plasmid). The SQ20B cells were treated with 2 μg/mL of doxycycline to induce TLR3 shRNA. (**e**) The expression of cancer stem cells (CSC) genes, epithelial-to-mesenchymal (EMT), and endogenous ligands genes and TLR2 in Detroit 562 cells after the stimulation of TLR3; ctrl adh—untreated adherent cells, ctrl sph—untreated spheres, pIC sph—poly (I:C)-treated spheres, pAU sph—poly (A:U)-treated spheres. * *p* < 0.05, ** *p* < 0.03, *** *p* < 0.01 (compared to the untreated adherent cells), # *p* < 0.05 (compared to the untreated spheres).

Previously, we established that the Detroit 562 cell line is a good model to study TLR3 function, as poly (I:C) treatment induced IL-6 secretion, and these cells responded to poly (I:C) by inducing apoptosis, which shows that TLR3 is functional [21]. Firstly, we have shown that Detroit 562 cells form tumor spheres when grown in low-adherence dishes and in special medium without serum but with the addition of FGF, EGF, and B27. However, we also noticed that the number of spheres grown after the treatment with poly (A:U) increased in comparison to the spheres grown only in the medium. Poly (A:U) is a more specific TLR3 ligand, as it does not activate other dsRNA receptors such as MDA5 and RIG-I. This was especially visible in the group where we counted larger spheres (≥300 μm), where 82 large spheres were present in poly (A:U)-treated spheres compared to only 47 in the control (untreated spheres, Figure 1a). We also observed this for FaDu cells (Figure S4). Additionally, we have shown here that the spheres not only adhere to one another, producing bigger spheres, but the number of cells also increases over time (Figure 1b). ALDH activity was elevated in tumor spheres compared to the adherent cells, and it was even higher in poly (A:U)-treated spheres, where the enhancement was also statistically significant compared to the untreated tumor spheres (Figure 1c). SQ20B-shcontrol cells, which are stably transfected with control plasmid, generate normal tumor spheres of circular shape, and SQ20B-shTLR3 cells, in which TLR3 is conditionally knocked down, do not produce tumor spheres, but cells stay rather scattered (Figure 1d).

We have further demonstrated that certain stemness markers (CD133, Oct4, and Sox) and mesenchymal markers (fibronectin and vimentin) were significantly increased after TLR3 stimulation (Figure 1e). Poly (I:C) induced the expression of DAMPs (endogenous ligands), especially S100A8 and S100A9, which were increased in a statistically significant manner even when compared to control spheres. The expression of TLR2 was also increased after poly (I:C) treatment compared to adherent cells and tumor spheres. Similar results were obtained for FaDu cells (Figure S5). Given the more pronounced changes observed in Detroit 562 cells, this cell line was selected for further investigation.

### 3.2. TLR3 Activation Induces the Expression of DAMPs and Their Release into the Microenvironment

**Figure 2:**
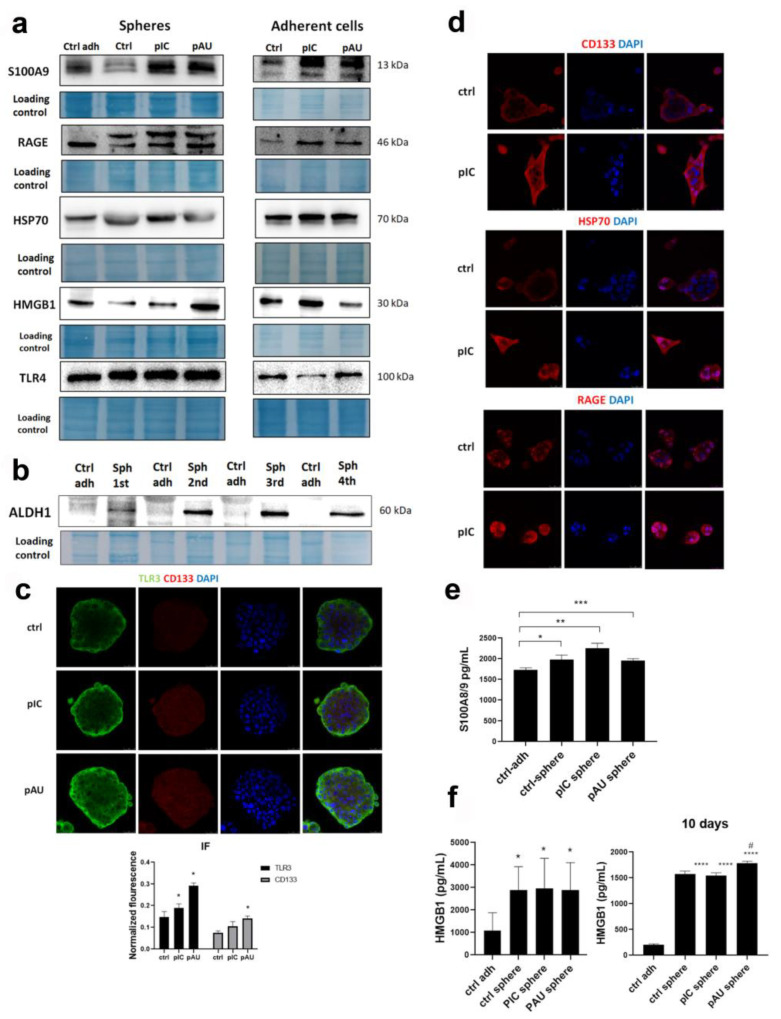
TLR3 induces the expression of damage-associated molecular patterns (DAMPs). (**a**) The expression of S100A9, RAGE, HSP70, HMGB1, and TLR4. ctrl adh—untreated adherent cells, pIC (cells/spheres treated with poly (I:C)), pAU (cells/spheres treated with poly (A:U). (**b**) ALDH1 expression through cancer stem cell generations. ctrl adh—untreated adherent cells, Sph 1st (spheres in the first generation), Sph 2nd (spheres in the second generation), Sph 3rd (spheres in the third generation), Sph 4th (spheres in the fourth generation). (**c**) Immunofluorescence of tumor spheres shows TLR3 and CD133 co-localization and expression. * *p* < 0.03 (compared to the untreated control) (C). (**d**) Immunofluorescence shows the expression of CD133, HSP70, and RAGE in adherent cells treated with poly (I:C). (**e**) ELISA shows the secretion of S100A8/9 in untreated adherent cells, untreated tumor spheres, and tumor spheres treated with poly (I:C) and poly (A:U). * *p* < 0.05, ** *p* < 0.03, *** *p* < 0.01 (compared to the adherent control cells). (**f**) ELISA shows the secretion of HMGB1 in untreated adherent cells, untreated tumor spheres, tumor spheres treated with poly (I:C) and poly (A:U), after 4 days (left) and after 10 days (right) of cultivating spheres prior to the treatment. * *p* < 0.05, ** *p* < 0.03, *** *p* < 0.01 (compared to the adherent control cells) * *p* > 0.05, **** *p* < 0.001 (compared to the adherent control cells), # *p* < 0.05 (compared to the untreated spheres).

We have confirmed here that TLR3 activation, either by poly (I:C) or poly (A:U), induced the expression of all tested DAMPs in adherent cells but also in tumor spheres of Detroit 562 cells. S100A9 expression was most prominently increased after TLR3 activation by both ligands in spheres and in adherent cells. RAGE and HSP70 expressions were moderately increased following TLR3 induction. HMGB1 was only increased when tumor spheres were treated with poly (A:U) and when adherent cells were treated with poly (I:C). We also tested TLR4, and it was slightly increased in tumor spheres compared to adherent cells (Figure 2a). Additionally, we followed ALDH1 expression across 4 generations of spheres and demonstrated that its expression is highest in the 2nd and 3rd generations of Detroit 562 tumor spheres (Figure 2b).

As shown by immunofluorescence in tumor spheres, TLR3 expression is stronger after the treatment with poly (I:C) and poly (A:U), as well as the expression of CD133, and TLR3 and CD133 co-localize (Figure 2c). Hence, we have confirmed the qPCR results. In adherent cells, CD133 and several DAMPS (HSP70 and RAGE) are upregulated after poly (I:C) treatment (Figure 2d).

ELISA analysis revealed that tumor spheres released significantly more S100A8/9 and HMGB1 into the tumor microenvironment than adherent cells. Poly (I:C) treatment induced a higher release of S100A8/9 compared to both controls; however, it was not statistically significant (Figure 2e). HMGB1 release from tumor spheres was almost 3 times higher compared to the adherent cells. Initially, HMGB1 release was similar in control and TLR3-activated spheres. However, after 10 days of sphere culture, TLR3 activation by poly (A:U) led to a statistically significant increase in HMGB1 release (Figure 2f).

### 3.3. TLR3 Activation Increases Cell Migration, Which Can Be Abolished by Aspirin and Metformin

Previously, we have shown that TLR3 activation induced the migration of Detroit 562 cells [7]. Here, we further investigated whether CSCs might release some factors into the tumor microenvironment that can increase the migration of surrounding tumor cells. The migration experiments revealed that even untreated (control) tumor spheres increase migration, which is further augmented by poly (I:C) treatment. We sought to determine whether certain DAMP inhibitors could attenuate poly (I:C)-induced migration. Therefore, we tested aspirin (HMGB1 inhibitor) [22], metformin (RAGE receptor inhibitor) [23], paquinimod (S100A8/A9 inhibitor) [24], and kahweol (HSP70 inhibitor) [25]. Aspirin (acetylsalicylic acid, ASA) is a well-known non-steroidal anti-inflammatory drug, metformin (MF) is a renowned drug for the treatment of type 2 diabetes, paquinimod (PAQ) is a drug that was developed to treat an autoimmune disease systemic lupus erythematosus, and kahweol (KW) is a diterpene found in Arabica coffee.

**Figure 3:**
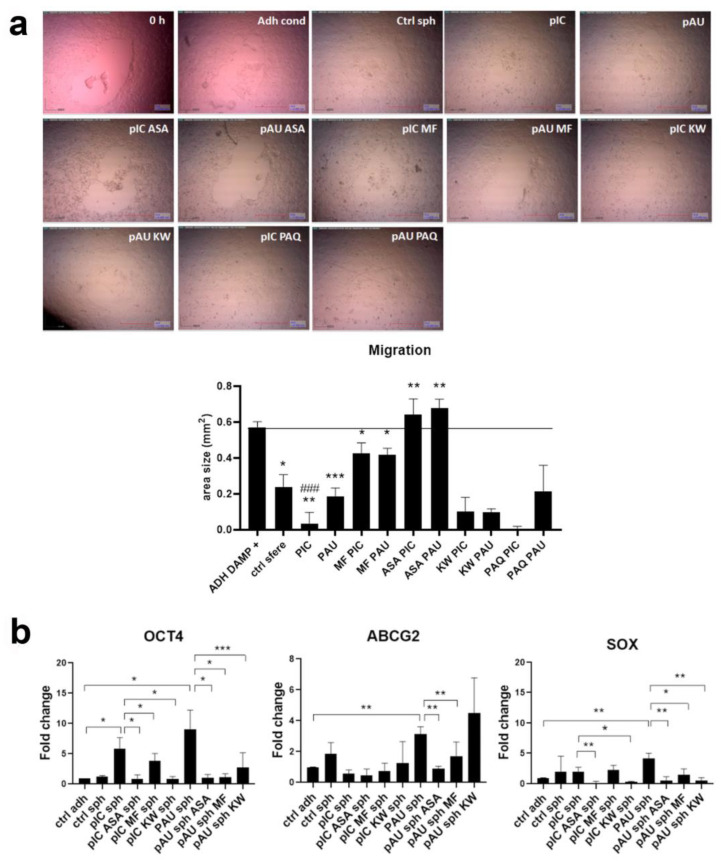
Tumor spheres and TLR3-activated tumor spheres release in the microenvironment factors that increase the migration and stemness of the adherent Detroit 562 cells, which can be abrogated by DAMP inhibitors. (**a**) Adherent cells were treated with the conditioned supernatant from untreated adherent cells (Adh cond) which served as a control, untreated tumor spheres (Ctrl sph), and tumor spheres treated with poly (I:C) (pIC), poly (A:U) (pAU), with poly (I:C) and acetylsalicylic acid (ASA) (pIC ASA), with poly (A:U) and acetylsalicylic acid (ASA) (pAU ASA), with poly (I:C) and metformin (MF) (pIC MF), with poly (A:U) and metformin (MF) (pAU MF), with poly (I:C) and kahweol (KW) (pIC KW), with poly (A:U) and kahweol (KW) (pAU KW), with poly (I:C) and paquinimod (PAQ) (pIC PAQ), and with poly (A:U) and paquinimod (PAQ) (pAU PAQ). * *p* < 0.05, ** *p* < 0.01, *** *p* < 0.00 (compared to adherent control). (**b**) The expression of OCT4, ABCG2, and SOX2 in tumor spheres treated with poly (I:C), and poly (A:U), but also the inhibitors of DAMPs. Ctrl adh—untreated adherent cells, ctrl sph—untreated tumor spheres, pIC sph—tumor spheres treated with poly (I:C), pIC ASA sph (tumor spheres treated with poly (I:C) and ASA), pIC MF sph (tumor spheres treated with poly (I:C) and MF), pIC KW sph (tumor spheres treated with poly (I:C) and KW), pAU sph—tumor spheres treated with poly (A:U), pAU ASA sph (tumor spheres treated with poly (A:U) and ASA), pAU MF sph (tumor spheres treated with poly (A:U) and MF), pAU KW sph (tumor spheres treated with poly (A:U) and KW). * *p* < 0.05, ** *p* < 0.03, *** *p* < 0.01 (compared to adherent cells). ### *p* < 0.05 (compared to the untreated tumor spheres).

The addition of metformin to the poly (I:C)-treated spheres abrogated its pro-migratory effect. This was even more prominent when ASA was added to the poly (I:C)-treated spheres, where the migration was reduced to the level of control. With the addition of kahweol and paquinimod, we did not observe this effect (Figure 3a). Additionally, by qPCR, we have shown that poly (I:C) and poly (A:U) treatment of the tumor spheres induces stemness marker *OCT4* expression, which is abrogated by the addition of ASA, MF, and KW. TLR3 activation in tumor spheres by poly (A:U) induced *ABCG2*, a marker of drug resistance, which was reduced by the addition of ASA and MF. Poly (A:U) treatment of tumor spheres induces the expression of *SOX2*, which was reduced by the treatment with ASA, MF, and KW (Figure 3b).

### 3.4. Immunohistochemistry of Patients’ HNSCC Tissue Shows Strong Expression of TLR3 Which Co-Localizes with CD133, ALDHA1, and DAMPs

To investigate the co-localization of TLR3 with stem cell markers (ALDH1A1 and CD133) and endogenous ligands (HSP70 and RAGE), we performed immunohistochemical analysis with fluorescently labeled antibodies on seven tumor tissue samples. These patients’ necrotic aspirates were previously collected and analyzed in HEKBlue cells, demonstrating TLR3 activation by necrotic aspirates from HNSCC patients [10]. The main purpose of histological samples in this study was to provide ex vivo proof of the co-localization of TLR3, DAMPs, and CSCs. We hypothesized that tumor cells that die by necrosis after therapy or due to lack of oxygen/nutrients can release endogenous ligands (DAMPs) into the microenvironment that can stimulate the self-renewal and maintenance of tumor stem cells, and possibly even their initiation.

**Figure 4:**
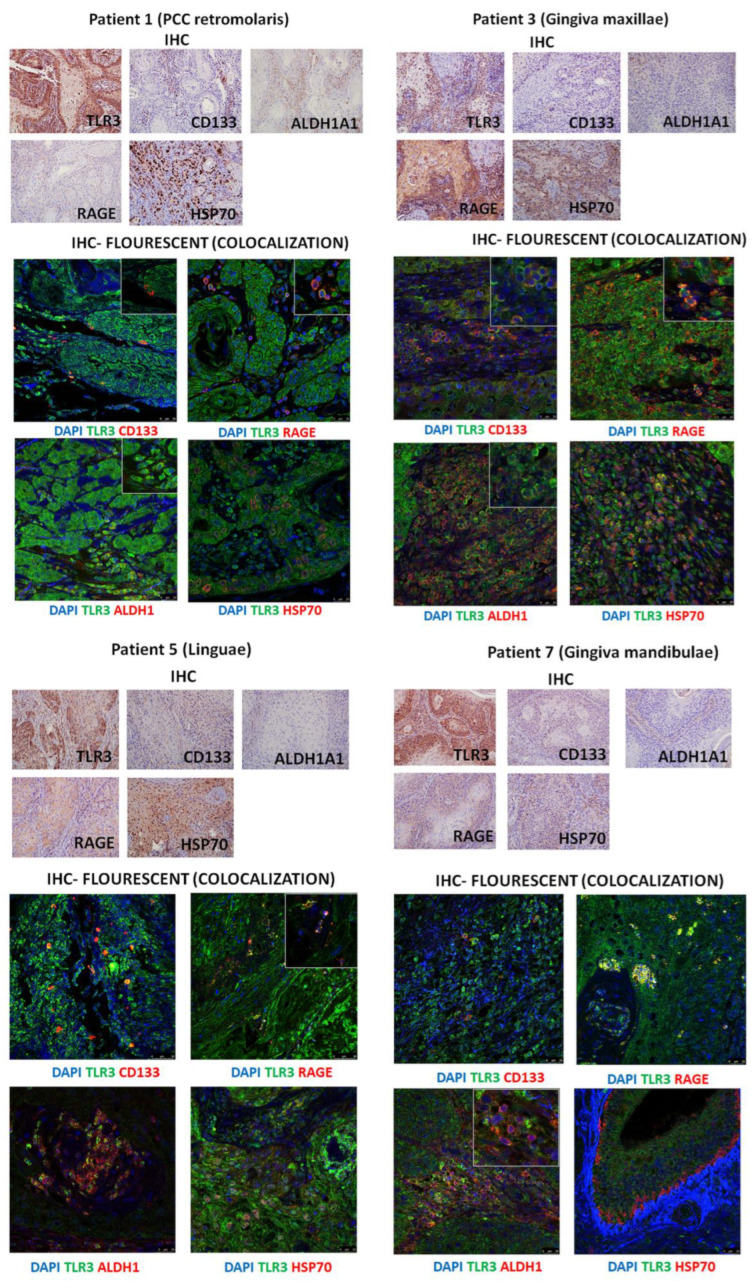
The expression of TLR3, CD133, ALDH1A1, RAGE, and HSP70 in different types of HNSCC patients’ tissues performed by standard immunohistochemistry and co-localization of TLR3 with CD133, ALDH1A1, RAGE, and HSP70 in different types of HNSCC patients’ tissues performed by fluorescent immunohistochemistry.

TLR3 was strongly expressed throughout the tissue in all patients, as shown in patient samples analyzed by regular immunohistochemistry, and also in samples analyzed by fluorescent immunohistochemistry which also allowed us to assess co-localization (Figure 4). CD133 appeared to be expressed in a small subset of cells randomly scattered throughout the cancer tissue, suggesting these cells might be cancer stem cells. CD133 co-localized with TLR3 in all cells and was expressed in all tested HNSCC cancer tissue (PCC retromolaris, gingiva mandibulae, gingiva maxillae, and linguae). ALDH1 was strongly expressed in lingual and gingiva maxillae and mandibulae tissue and it strongly co-localized with TLR3. RAGE was strongly expressed in the gingiva maxillae throughout the cancer tissue; however, in other tested tissues, RAGE was expressed in some cells where it co-localized strongly with TLR3. HSP70 was strongly expressed in lingual, PCC retromolaris, and gingiva maxillae and strongly co-localized with TLR3 in this tissue. All our IHC results undoubtedly demonstrate that TLR3 expression co-localizes with cancer stem markers (CD133 and ALDH1) and tested DAMPs (RAGE and HSP70). The clinical characteristics of the tested patients are presented in Table 1. Almost all tested patients showed features of advanced cancer.

### 3.5. Gamma Irradiation in Combination with Aspirin and Poly (I:C) Reduces the Survival of Adherent Tumor Cells

**Figure 5:**
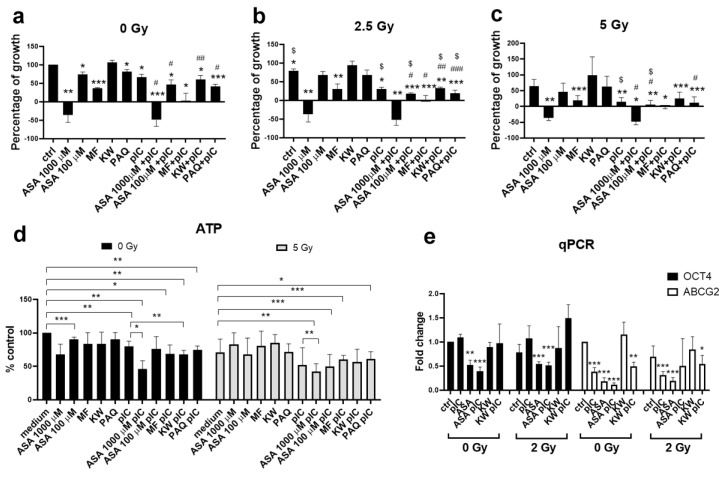
Comparison of radiotherapy by γ-rays in combination with poly (I:C) and DAMP inhibitors on adherent cells and tumor spheres treated for 72 h. (**a**) The viability of adherent Detroit 562 cells treated with poly (I:C) and DAMP inhibitors without the irradiation performed by MTT assay (ctrl adh—untreated adherent cells, ASA—cells treated with acetylsalicylic acid, MF—cells treated with metformin, KW—cells treated with kahweol, PAQ—cells treated with paquinimod, pIC—cells treated with poly (I:C), ASA+pIC—cells treated with ASA and poly (I:C), MF+pIC—cells treated with MF and poly (I:C), KW+pIC—cells treated with KW and poly (I:C), PAQ+pIC—cells treated with PAQ and poly (I:C)). (**b**) The viability of adherent Detroit 562 cells treated with poly (I:C) and DAMP inhibitors followed by irradiation with 2.5 Gy performed by MTT assay. (**c**) The viability of adherent Detroit 562 cells treated with poly (I:C) and DAMP inhibitors followed by irradiation with 5 Gy performed by MTT assay. (**d**) The viability of Detroit 562 tumor spheres treated with poly (I:C) and DAMP inhibitors, either without irradiation (0 Gy) or in combination with irradiation (5 Gy) performed by Vialight assay. (**e**) The expression of OCT4 and ABCG2 performed by qPCR in tumor spheres treated with poly (I:C) and DAMP inhibitors without irradiation (0 Gy), or in combination with irradiation with 2 Gy. * *p* < 0.05, ** *p* < 0.01, *** *p* < 0.005 (compared to the control cells); # *p* < 0.05, ## *p* < 0.01, ### *p* < 0.005 compared to DAMP inhibitor treatment), $ *p* < 0.05 (compared to non-irradiated cells).

Poly (I:C) alone has a radiosensitizing effect, as we have previously published [26]. We first tested the radiosensitizing effect of poly (I:C), DAMP inhibitors, and their combination in adherent Detroit 562 cells. In this study, the highest concentration of ASA (1000 μM) used on the adherent cells was too excessive (Figure S6), but we included it because tumor spheres were less sensitive, as expected, since cancer stem cells’ main feature is chemo- and radio-resistance. The results showed that metformin reduced cell survival by 60%, while in combination with poly (I:C), the effect was even more prominent, resulting in the complete elimination of the cells. Additionally, ASA in the concentration of 100 μM, paquinimod, and kahweol in combination with poly (I:C) were effective (Figure 5a). A dose of 2.5 Gy slightly reduced cell survival. However, in combination with poly (I:C), and especially in the combination of poly (I:C) and DAMP inhibitors, it was very effective, and only 20–30% of cells survived (depending on the combination) (Figure 5b). A 5 Gy dose inhibits cell growth by 40%. Combining this with poly (I:C) significantly enhances the effect, reducing cell survival to 10–20%, depending on the specific combination of DAMP inhibitors used. Furthermore, the combinations of 100 μM ASA with poly (I:C) and MF with poly (I:C) proved particularly effective, resulting in complete cell death (Figure 5c). Next, we examined the survival of tumor spheres following the treatment with poly (I:C), DAMP inhibitors, and 5 Gy irradiation (Figure 5d). A dose of 5 Gy reduced the survival of tumor spheres by only 30%, while a dose of 1000 μM ASA reduced survival by only 35%. However, the results showed that the combined treatment of ASA with pIC, even without irradiation, reduced the survival of spheres by more than 50%, while irradiation did not significantly increase this effect. The combination of poly (I:C) and irradiation reduced survival by almost 50%. ASA in combination with poly (I:C) and irradiation was not significantly more effective than poly (I:C) alone. Finally, we examined the expression of the stemness marker *OCT4* and the drug resistance marker *ABCG*2 after the treatment of tumor spheres with all these combinations of drugs and irradiation (Figure 5e). We chose a dose of 2 Gy to compare the results with proton irradiation, where we also used a dose of 2 Gy, but in that case was very effective. We showed that the treatment with ASA alone reduces the expression of the *OCT4* gene by 50%, which was slightly increased by the addition of poly (I:C), but not statistically significant. Combined treatment with radiation does not significantly affect the expression of *OCT4* compared to the expression without irradiation. However, the combined treatment of poly (I:C) and ASA almost completely reduced *ABCG2* expression, although poly (I:C) and ASA alone are effective and reduced *ABCG2* expression by 50% and 80%, respectively. Radiation in the case of poly (I:C) and ASA when applied individually does not significantly affect the *ABCG2* expression, but in the case of the combination of ASA and poly (I:C), it increased the expression, suggesting that ASA or poly (I:C) alone or applied together but without radiotherapy are a better therapeutic option.

### 3.6. The Combination of Aspirin and Proton Irradiation Effectively Eradicates Cancer Stem Cells

**Figure 6:**
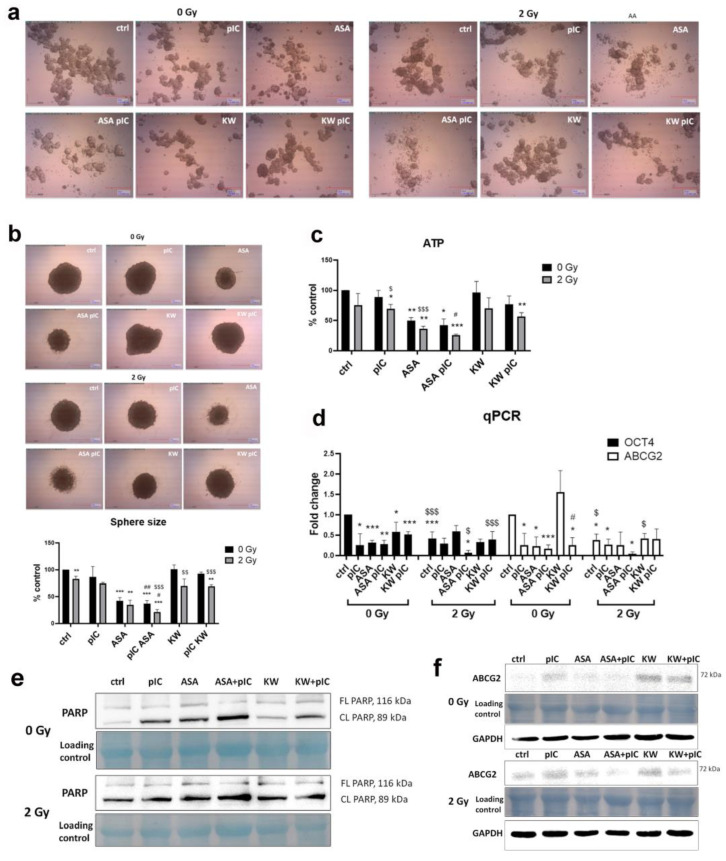
Radiotherapy of tumor spheres by proton irradiation. (**a**) The morphology of tumor spheres after the treatment for 72 h with poly (I:C) and DAMP inhibitors without the irradiation (0 Gy), or followed by proton therapy (2 Gy). Ctrl—untreated tumor spheres, pIC—tumor spheres treated with poly (I:C), ASA—tumor spheres treated with acetylsalicylic acid, ASA+pIC—tumor spheres treated with ASA and poly (I:C), KW—tumor spheres treated with kahweol, KW+pIC—tumor spheres treated with KW and poly (I:C). (**b**) The size and the morphology of a single tumor sphere treated with poly (I:C) and DAMP inhibitors without the irradiation (0 Gy), or followed by proton therapy (2 Gy). (**c**) The viability of tumor spheres after the treatment with poly (I:C) and DAMP inhibitors without the irradiation (0 Gy), or followed by proton therapy (2 Gy) performed by Vialight assay. (**d**) The expression of OCT4 and ABCG2 performed by qPCR in tumor spheres treated with poly (I:C) and DAMP inhibitors without irradiation (0 Gy), or in combination with proton therapy with 2 Gy. (**e**) PARP cleavage detected by Western blot after the treatment of tumor spheres with poly (I:C) and DAMP inhibitors without irradiation (0 Gy), or in combination with proton therapy with 2 Gy). (**f**) ABCG2 expression detected by Western blot after the treatment of tumor spheres with poly (I:C) and DAMP inhibitors without irradiation (0 Gy), or in combination with proton therapy with 2 Gy. Loading control membrane dyed with amido black; FL—full length, CL—cleaved PARP. * *p* < 0.05, ** *p* < 0.01, *** *p* < 0.001 (compared to the control), # *p* < 0.05, ## *p* < 0.03 (compared to ASA or KW treatment alone), $ *p* < 0.05, $$ *p* < 0.01, $$$ *p* < 0.001 (irradiated cells compared to the non-irradiated).

Irradiation of cells was conducted within innovative, custom-built Teflon chambers, fully described in Supplementary Data (Figures S1–S3). In this set of the experiments, we have shown that ASA alone, and in combination with poly (I:C), changes sphere morphology (Figure 6a), reduces sphere size (Figure 6b), reduces the survival (Figure 6c), and decreases the expression of stemness marker *OCT4*, and drug resistance marker *ABCG2* (Figure 6d). However, proton irradiation enhanced the cytotoxic effects of both ASA and the combination of ASA with poly (I:C), resulting in only 20–30% cell survival. Moreover, proton irradiation alone reduced the expression of *OCT4* and *ABCG2* by more than 50%, and with the addition of ASA and poly (I:C), no residual expression was detected. Finally, we have shown that apoptosis is the mechanism behind this effect, as poly (I:C), ASA, ASA with poly (I:C), and KW with poly (I:C) induce PARP cleavage even without proton therapy. Following proton therapy, cleaved PARP expression was observed in control spheres and across all treatment groups (Figure 6e). The expression of ABCG2 protein was also reduced after the treatment with the combination of ASA and poly (I:C) without and with proton irradiation (Figure 6f). We conclude here that proton therapy is a better choice for tumor cell treatment, as the same dose is more efficient than γ-irradiation and it is also more specific, as it deposits most of its energy right at the tumor site, which reduces the effect of the irradiation on surrounding normal tissue.

## 4. Discussion

We demonstrate here that TLR3 activation promotes stemness in HNSCC cells, as evidenced by larger tumor sphere formation, upregulation of stemness- and EMT-related genes, and elevated ALDH activity. To our knowledge, there has been only one study that associated TLR3 with CSCs, but it was conducted on breast cancer [27]. The authors showed that β-Catenin and NF-κB are the key players that contribute to stemness, which were activated after TLR3 stimulation. There is also one study that revealed TNF receptor-associated factor 6 (TRAF6) as a key player in tumor metastasis regulation through EMT and CSC phenotypes [28]; however, as TRAF6 is the signaling molecule in the TLR3 signaling pathway, a similar effect can be generated with TLR3 stimulation. Pries et al. showed that TLR3 is overexpressed in 80% of solid tumors and all HNSCC cell lines, but not in the healthy tissue surrounding the tumor. We have previously shown that TLR3 may play a role in tumor metastasis. There were notable differences in TLR3 expression and functionality between primary and metastatic HNSCC carcinoma cell lines [5]. Umemura et al. (2012) compared primary and metastatic HNSCC cell lines and found that metastatic cell lines are especially sensitive to TLR3-TRIF-NK-κB-induced cell death [29]. Activation of TLR3 can also trigger metabolic reprogramming in HNSCC cell lines, resulting in enhanced aerobic glycolysis consistent with the Warburg effect [7]. Veyrat et al. (2016) also showed that HNSCC cell lines treated with poly (A:U) activate TLR3, which increases HIF-1α protein expression and facilitates metabolic shift [6].

Our findings also demonstrate that TLR3 activation induces DAMP expression. Since TLR3 agonists, such as dsRNA fragments, may be consistently present in the HNSCC tumor microenvironment and necrotic fluids, these endogenous DAMPs may create a positive feedback loop by further activating TLR3, potentially driving a pro-tumorigenic effect, as we have previously reported [10]. No external signals, such as viral infection or injury, are required to induce TLR3 activation. Common tumor microenvironment conditions, such as hypoxia or starvation, are sufficient. Our immunofluorescence analysis showing the co-localization of TLR3, DAMPs, and stemness markers in patient tissue supports this hypothesis. We have also shown that TLR3 activation induces HNSCC cell migration, a process that can be hindered by inhibiting endogenous ligands, particularly with aspirin as an HMGB1 inhibitor. Even though poly (I:C) and poly (A:U) (more specific TLR3 activator) showed different effects in some of the experiments, poly (A:U) was undoubtedly superior in all the important discoveries. It induced high *CD133*, *FN1*, and HMGB1 expression, stimulated the secretion of HMGB1, and induced the expression of *OCT4*, *ABCG2,* and *SOX2,* which was abolished by the treatment with DAMP inhibitors.

It has also been previously shown that the activation of different TLRs may increase the expression of other TLRs. Such an example is necrosis-induced TLR3, which may promote TLR2 expression in gingival cells [30]. Therefore, we are not exploring only the role of TLR3 in DAMP-mediated CSCs’ contribution to tumor progression, but possibly also other TLRs as well. Di Lorenzo et al. showed recently that doxorubicin can induce the release of HMGB1. This can activate TLR2 signaling in tumor cells, leading to the development of a chemotherapy-resistant phenotype [31], and it has previously been shown that TLR2 and HMGB1 have an important role in the self-renewal of breast CSCs [32]. Since aspirin is an HMGB1 inhibitor, and we have shown that TLR3 activation induces TLR2 expression, it is possible that the observed effect of the HMGB1 inhibitor on tumor sphere survival is linked to TLR2 and its inability to be activated with its ligand. Finally, it has been previously reported that ASA can induce apoptosis. Recent work in Caenorhabditis elegans [33] confirmed these findings, demonstrating aspirin’s tumor-suppressive and radio/chemo-sensitizing effects. In a recent study, it was shown that ASA can inhibit Jurkat cell proliferation and induce apoptosis by downregulating T cell immunoglobulin and ITAM domain (TIGIT) expression and the anti-apoptotic B cell lymphoma 2 (BCL2) protein, while upregulating BCL2-associated X protein (BAX) expression [34]. Furthermore, Zhang et al. found that aspirin promoted G1/S cell cycle arrest and confirmed that aspirin induces apoptosis in a concentration-dependent manner in colon cancer cell lines [35]. In melanoma and hepatocellular carcinoma, ASA also has anti-cancer properties and promotes apoptosis [36,37]. Aspirin can block glycolysis to inhibit non-small cell lung cancer cell proliferation [38]. Resania et al. showed that ASA can boost the effect of miR-302/367 to reverse the epithelial/mesenchymal transition and inhibit the invasion, migration, and angiogenesis in breast cancer cells [39]. ASA can also suppress breast cancer metastasis to the lung by targeting anoikis resistance [40] and inhibiting the hypoxia-mediated stemness of lung cancer cells [41]. The combined use of ASA with chemotherapeutic agents or other drugs is also being explored. Susan et al. showed a synergistic cytotoxic effect of ASA and 5-fluorouracil on colon carcinoma cells [42]. Dipyridamole, an antiplatelet drug, augments the anti-cancer ability of aspirin against colorectal cancer by inducing apoptosis [43]. Aspirin enhances the therapeutic effectiveness of gemcitabine in treating the pancreatic cancer [44], enhances cisplatin sensitivity in ovarian cancer [45], and improves the synergistic effect of EGFR inhibitor and anti-mitotic chemotherapeutic Vinorelbine on lung cancer cells [46]. Jiang et al. showed that the mechanism of aspirin enhancement of the sensitivity of colon cancer cells to cisplatin is by abolishing the binding of NF-kappaB to the COX-2 promoter [47]. The potential impact of ASA on CSCs is also under investigation, but the number of studies is limited. Zou et al. revealed that ASA enhances the cisplatin therapy in esophageal squamous cell carcinoma by inhibiting CSCs [48]. Another study showed that ASA has an inhibitory effect on colorectal cancer patient-derived spheroids [49]. Aspirin also inhibits the growth of glioblastoma multiforme and CSC properties [50]. Additionally, aspirin blocks tumor-initiating cells [51] and the acquisition of chemoresistance in breast cancer by disrupting an NFκB-IL6 signaling axis [52]. The cumulative evidence strongly supports the anti-tumorigenic properties of aspirin. The main advantage of aspirin is that it is an inexpensive, well-tolerated, and easily accessible drug, often used daily in patients with cardiovascular problems, and there are a number of studies showing that long-term use of aspirin reduces the occurrence of cancer or metastasis [53,54,55,56,57]. In breast cancer, aspirin may reduce the incidence of metastasis in patients who have previously received anti-cancer therapies [58]. In a pre-clinical model of breast cancer, aspirin delayed time to metastasis [59]. There is even one ongoing clinical trial trying to determine whether adjuvant therapy with low-dose aspirin can enhance disease-free survival in patients who have undergone treatment for colorectal cancer liver metastases [60]. However, aspirin had no beneficial effect in older patients with cancer [61]. We propose that the anti-tumor and anti-metastatic effects of aspirin are mediated, at least in part, by its impact on cancer stem cells, which are critical drivers of tumor initiation and metastasis.

γ-irradiation therapy in combination with poly (I:C) and DAMP inhibitors was an adequate therapeutic option for adherent cancer cells, but it was not effective on CSCs. It may be that tumor spheres activate certain cell signaling pathways, which allows them to induce mechanisms of radio-resistance, as has been proposed previously [62,63,64]. On the other hand, proton therapy proved to be the better option as it was quite effective on cancer spheres by inducing apoptosis, especially in combination with ASA alone, or in combination with poly (I:C) and ASA. A similar result was previously shown in non-small cell lung cancer. The authors demonstrated that compared with photons, protons caused significantly lower cell viability [65]. In a systematic review published recently, the authors searched through the databases for articles on CSCs irradiated by charged particles. While CSCs exhibited radio-resistance compared to non-CSCs, particle irradiation, alone or in combination with drugs, effectively overcame this resistance, reducing proliferation, invasion, and migration, and inducing DNA double-strand breaks (DSBs) that were more difficult to repair [66]. Proton beams generally produce a higher relative biological effectiveness (RBE) compared to photons (like X-rays or gamma rays). Proton beam therapy is gaining attention as a valuable option for HNSCC treatment, especially for tumors located above the hyoid bone, where the anatomy is particularly complex. This approach with proton beam treatment could especially benefit in sensitive areas, as it can better protect and avoid surrounding sensitive structures [67]. For patients with recurrent disease, proton therapy might be the go-to curative option. One of its biggest advantages is that it allows delivery of a strong dose of radiation to the tumor without harming the healthy tissue that was already exposed during earlier treatments. However, reirradiation of the area presents certain difficulties. Some studies show that although this method can be effective in retargeting the tumor, there is also a risk of developing serious side effects like osteoradionecrosis, which affects the bone [68]. On the reassuring side, Mumaw et al. 2024 found that in patients who received proton therapy on just one side of the neck, the cancer rarely came back on the opposite side [69]. Although the number of studies using proton therapy as a therapeutic option for HNSCC is still limited, this kind of therapy appears to be an attractive radiotherapy modality with excellent treatment outcomes in patients with HNSCC [67]. Future studies should investigate the synergistic effects of combining proton therapy with ASA or poly (I:C).

An important question that remains unanswered is the precise mechanism by which these effects occur, and whether other TLR- and/or other signaling pathways are involved. Although our previous work [19] has shown that TLR3 activation can stimulate the expression of other stemness-related factors/proteins, further research is required to elucidate this process. One of the limitations of our study is the lack of in vivo confirmation, which would be crucial.

## 5. Conclusions

In conclusion, we have demonstrated that TLR3 might be an important player in CSC maintenance. Its activation also induced the expression and the release of DAMPs, important factors in tumor promotion and immune system evasion. In addition, our study demonstrates the promising potential of repurposing DAMP inhibitors, aspirin, and possibly metformin, for novel cancer therapies, particularly in combination with proton therapy to target CSCs. γ irradiation therapy in combination with aspirin or metformin might also be a good therapeutic option for cancer cells, but our results indicate it is not effective on CSCs. This study is the first, to our knowledge, to explore this combined approach.

## Figures and Tables

**Table 1:** Clinical features of the patients.

Patient No	pN Status	No. of Metastases	ENE	PVI	LVI	PNI	Bone Invasion	SPT	Histopathological Subtype	**TNM**	**HPV**	**Alcohol**	**Tobacco**
1	pN+	3	Yes	Yes	No	Yes	Yes	No	retromolar	T4aN0M0	No	Yes	Yes
2	pN0	0	No	No	No	No	No	No	gingiva mandibulae	T4aN3bM0	No	Yes	Yes
3	pN+	1	Yes	Yes	No	No	No	No	gingiva maxillae	T4aN0M0	No	Yes	Yes
4	pN+	3	Yes	No	No	Yes	No	No	linguae	T4aN3bM0	No	Yes	Yes
5	pN+	3	No	No	Yes	No	No	No	linguae	T4aN3bM0	No	Yes	Yes
6	pN0	0	No	No	No	Yes	Yes	No	retromolar	T3N2bM0	No	Yes	Yes
7	pN0	0	No	No	No	No	Yes	Yes	gingiva mandibulae	T4aN0M0	No	Yes	Yes

pN0—No regional lymph node metastases. pN+—Presence of regional lymph node metastases. ENE (Extranodal Extension)—Extranodal tumor spread beyond the lymph node capsule. PVI (Perivascular Invasion)—Tumor invasion into tissues surrounding blood vessels. LVI (Lymphovascular Invasion)—Presence of tumor cells within lymphatic or blood vessels. PNI (Perineural Invasion)—Tumor invasion along or within nerves. SPT (Secondary Primary Tumor)—Presence of a second independent primary tumor.

## Data Availability

The original contributions presented in this study are included in the article/Supplementary Materials. Further inquiries can be directed to the corresponding author.

## Supplementary Materials

The following supporting information can be downloaded at https://www.mdpi.com/article/10.3390/cancers17132157/s1: Table S1. Primer sequences. Table S2. List of primary antibodies. Table S3. List of secondary antibodies. Figure S1. The scheme of cell chamber used for proton irradiation. The chamber consists of 3 parts: the first part serves to hold the Mylar on the second part, and the third part serves as a cover. Cells grow attached to the collagen-coated Mylar. Side view (A) and view rotated for 45°. Figure S2. Chambers for cells during proton irradiation experiment and their position on the carrier. Figure S3. Cell chamber on the carrier during irradiation. A tube on the left delivers the proton beam, and a camera on the bottom right was used for chamber alignment. Figure S4. Number of spheres when FaDu cells were cultivated alone (ctrl) or with the addition of poly (I:C) (PIC) or poly (A:U) (PAU). Figure S5. TLR3 induces the expression of damage-associated molecular patterns (DAMPs), CSC genes, EMT genes, and TLR2 in FaDu cells. Figure S6. The titration of aspirin concentration by MTT assay after 48 h.

## Abbreviations

The following abbreviations are used in this manuscript:

ALDH	aldehyde dehydrogenase
ASA	acetylsalicylic acid
BAX	BCL2-associated X protein
BCL2	B cell lymphoma 2
CSCs	Cancer stem cells
DAMPs	damage-associated molecular patterns
DSB	DNA double-stranded breaks
EMT	epithelial-to-mesenchymal transition
HNSCC	head and neck squamous cell carcinoma
HSP70	heat shock protein 70
KW	kahweol
MF	metformin
PAQ	paquinimod
RAGE	receptor for advanced glycation end products
SAA	serum amyloid A
TIGIT	T cell immunoglobulin and ITAM domain
TLR3	Toll-like receptor 3
TRAF6	TNF receptor-associated factor 6
