# Supplementary material for: Targeting DAMPs by Aspirin Inhibits Head and Neck Cancer Stem Cells and Stimulates Radio-Sensitization to Proton Therapy
Source: Cancers (Basel). 2025 Jun 26;17(13):2157. doi: 10.3390/cancers17132157 (PMC12249109; doi:10.3390/cancers17132157)

Supplementary data

Table S1. Primer sequences

Gene	Forward primer	Reverse primer	Accession number
TLR2	5'-CAGGTGACTGCTCGGAGTTC-3'	5'-CACAACTACCAGTTGAAAGCAGTGA-3'	NM_003264
ABCG2	5′-TACCTGTATAGTGTACTTCAT-3'	5′-GGTCATGAGAAGTGTTGCTA-3'	NM_004827
HMGB1	5'-ATATGGCAAAAGCGGACAAG-3'	5'-AGGCCAGGATGTTCTCCTTT-3'	NM_002128
RAGE	5'-CCGAGTCCGTGTCTACCAGATT-3'	5'-CACATGTCCCCACCTTATTGG-3'	NM_001136
HSP70	5'-TGGAGTCCTACGCCTTCAAC-3'	5'-ATGGGGTTACACACCTGCTC-3'	NM_005345
S100A8	5'-GCTAGAGACCGAGTGTCCTCAG-3'	5'-GCCCATCTTTATCACCAGAATG-3'	NM_002965
S100A9	5'-TGGAGGACCTGGACACAAATG-3'	5'-TCGTCACCCTCGTGCATCTT-3'	NM_002966
Snail	5'-ACCACTATGCCGCGCTCTT-3'	5'-GGTCGTAGGGCTGCTGGAA-3'	NM_005985
Vimentin	5'-AATGGCTCGTCACCTTCGTGAAT-3′	5'-CAGATTAGTTTCCCTCAGGTTCAG-3′	NM_003380
ALDH1A1	5'-ACTGCTCTCCACGTGGCATCTTTA-3'	5'-TGCCAACCTCTGTTGATCCTGTGA-3'	NM_000689
ALDH1A3	5'-ACCTGGAGGTCAAGTTCACCAAGA-3'	5'-ACGTCGGGCTTATCTCCTTCTTCC-3'	NM_000693
Nanog	5'-CATGAGTGTGGATCCAGCTTG-3'	5'-CCTGAATAAGCAGATCCATGG-3'	NM_024865
Oct4	5'-GTGGAGGAAGCTGACAACAA-3'	5'-ATTCTCCAGGTTGCCTCTCA-3'	NM_002701
Sox2	5'-GAGCTTTGCAGGAAGTTTGC-3'	5'-GCAAGAAGCCTCTCCTTGAA-3'	NM_003106
CD133	5'-CAGAGTACAACGCCAAACCA-3'	5'-AAATCACGATGAGGGTCAGC-3'	NM_006017
CD44 standard	5′-TCCAACACCTCCCAGTATGACA-3′	5′-GGCAGGTCTGTGACTGATGTACA-3′	NM_000610
CD44-14v	5'-ATAGGAATGATGTCACAGGTGG-3'	5'-CGATTGACATTAGAGTTGGAATCTCC-3'	custom variant
E-cadherin	5′-GGAGGAGAGCGGTGGTCAAA-3′	5′-TGTGCAGCTGGCTCAAGTCAA-3′	NM_004360
Fibronectin	5′-GTGTGTTGGGAATGGTCGTG-3′	5′-GACGCTTGTGGAATGTGTCG-3′	NM_002026
28S RNA	5'-TTGAAAATCCGGGGGAGAG-3'	5'-ACATTGTTCCAACATGCCAG-3'	NR_003287

Table S2. List of primary antibodies

Target	Clone	Host species	Dilution	Manufacturer (cat. no.)
**HSP70**	**W27**	Mouse	1:1000 (WB); 1:100 (IHC, ICC)	Santa Cruz Biotechnology (sc-24)
**HMGB1**	**HAP46.5**	Mouse	1:1000 (WB)	Santa Cruz Biotechnology (sc-56698)
**S100A9**	**B-5**	Mouse	1:1000 (WB)	Santa Cruz Biotechnology (sc-376772)
**TLR4**	**25**	Mouse	1:1000 (WB)	Santa Cruz Biotechnology (sc-293072)
**RAGE**	**A-9**	Mouse	1:1000 (WB); 1:100 (IHC, IF, ICC)	Santa Cruz Biotechnology (sc-365154)
**ALDH1A1**	**H-4**	Mouse	1:1000 (WB); 1:100 (IHC, IF)	Santa Cruz Biotechnology (sc-374076)
**TLR3**	**polyclonal**	Rabbit	1:100 IHC, IF	ABCAM (ab62566)
**PARP**	**46D11**	Rabbit	1:1000 (WB)	Cell signaling technology (9532)
**CD133 (Prominin-1)**	**17A6.1**	Mouse	1:100 (IHC, IF, ICC)	Merck (MAB4399-I)
**ABCG2**	**BXP-21**	Mouse	1:100 (WB)	Santa Cruz Biotechnology (sc-58222)

Table S3. List of secondary antibodies

Host reactivity	Fluorophore/ Conjugate	Application	Dilution	Supplier (cat. no.)
Goat anti-Rabbit IgG	Alexa Fluor 555	IF, IHC		Invitrogen (A-21428)
Goat anti-Mouse IgG	Alexa Fluor 647	IF, IHC		Invitrogen (A-21235)
Anti-rabbit IgG	HRP conjugated	WB	1:3000	Cell Signaling Technology (7074)
Anti-mouse IgG	HRP conjugated	WB	1:5000	Amersham (NA931)

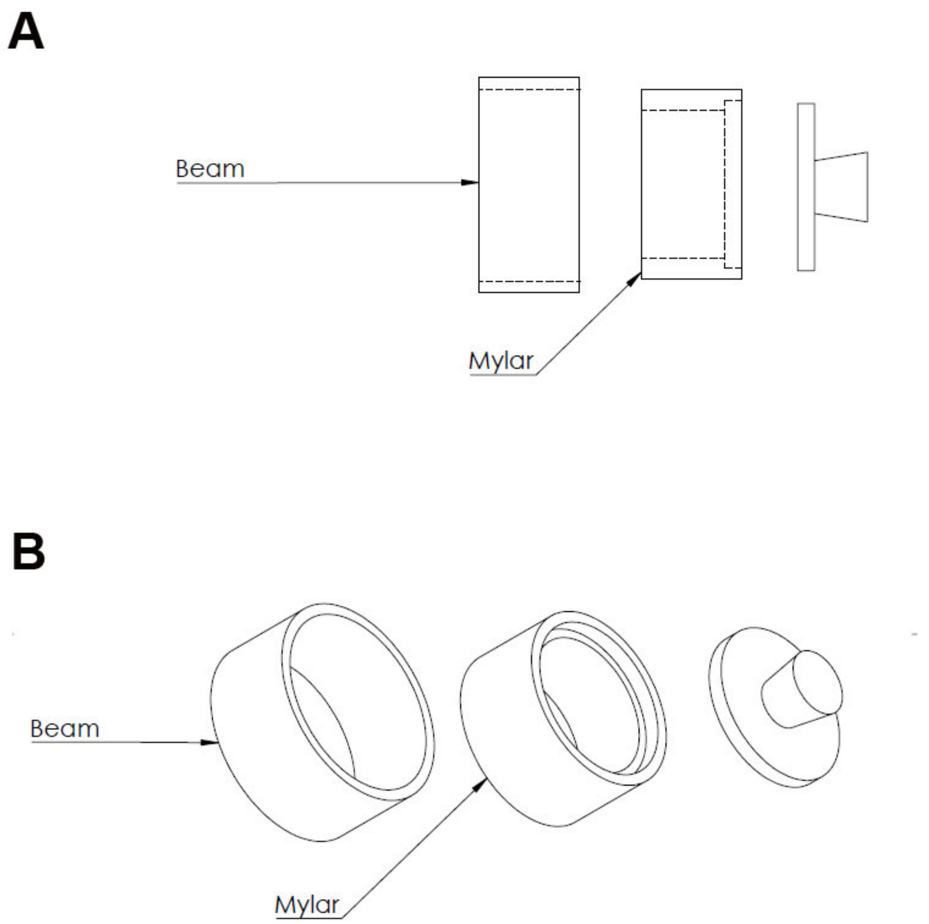

Figure S1. The scheme of cell chamber used for proton irradiation. The chamber consists of 3 parts: The first part serves to hold the Mylar on the second part, and the third part serves as a cover. Cells grow attached to the collagen-coated Mylar. Side view (A) and view rotated for 45°.

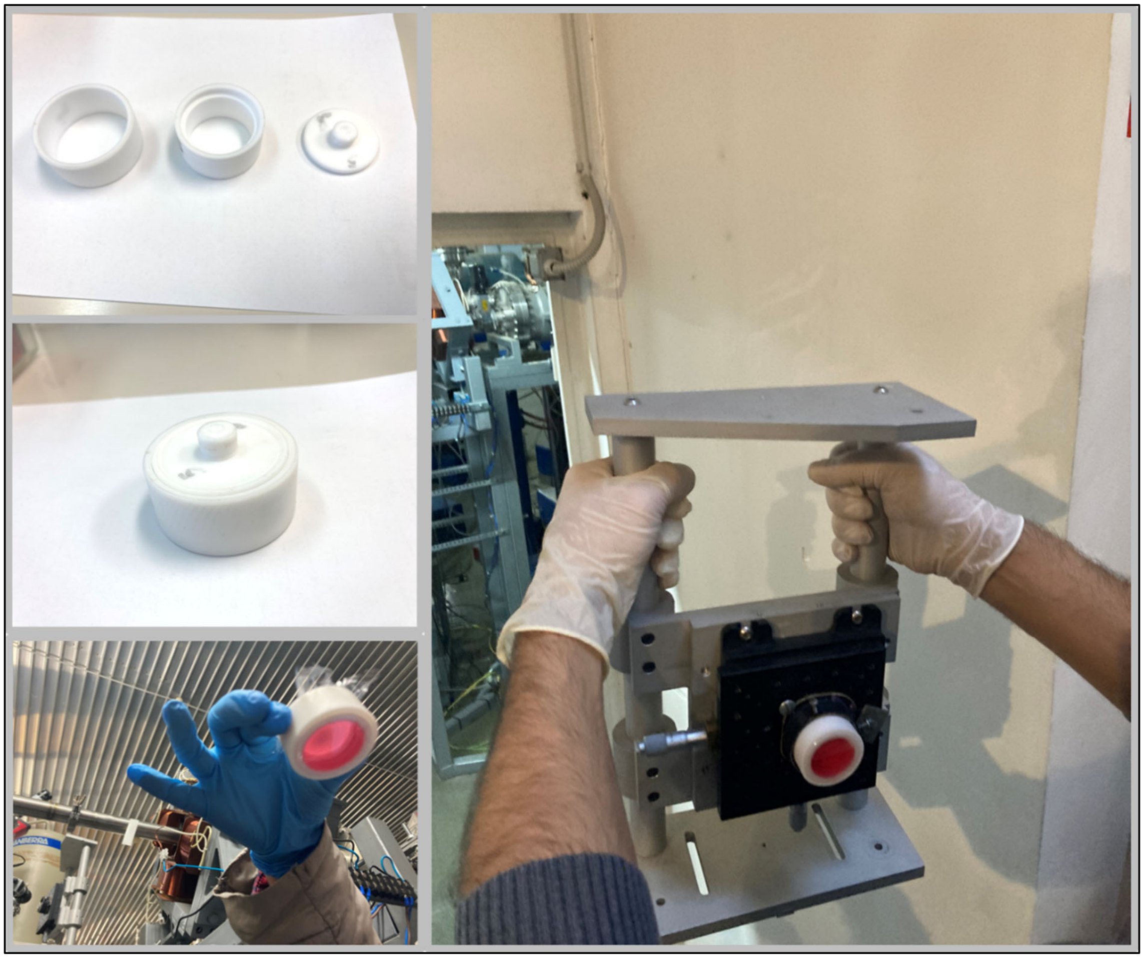

Figure S2. Chambers for cells during proton irradiation experiment, and their position on the carrier

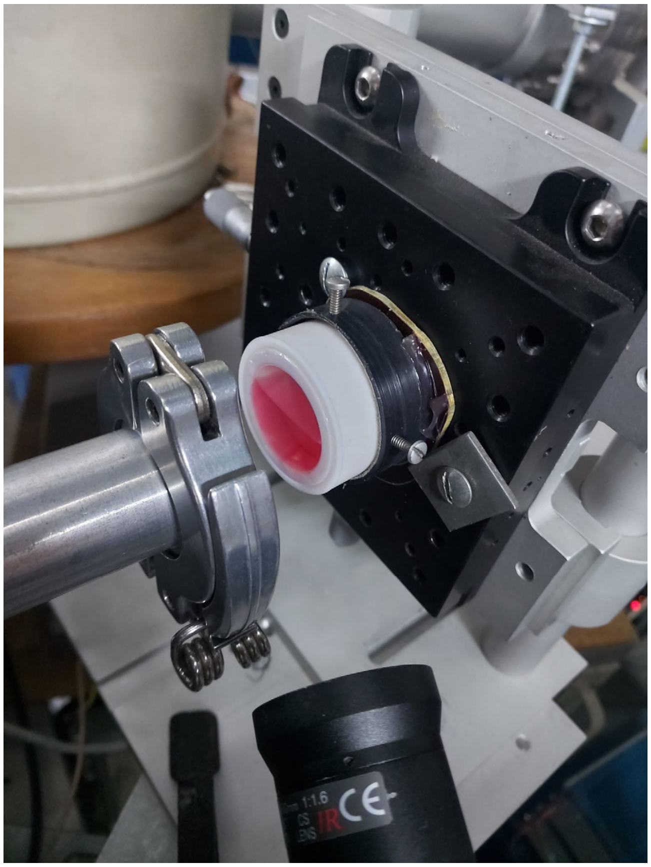

Figure S3. Cell chamber on the carrier during irradiation. A tube on the left delivers the proton beam, and a camera on the bottom right was used for chamber alignment.

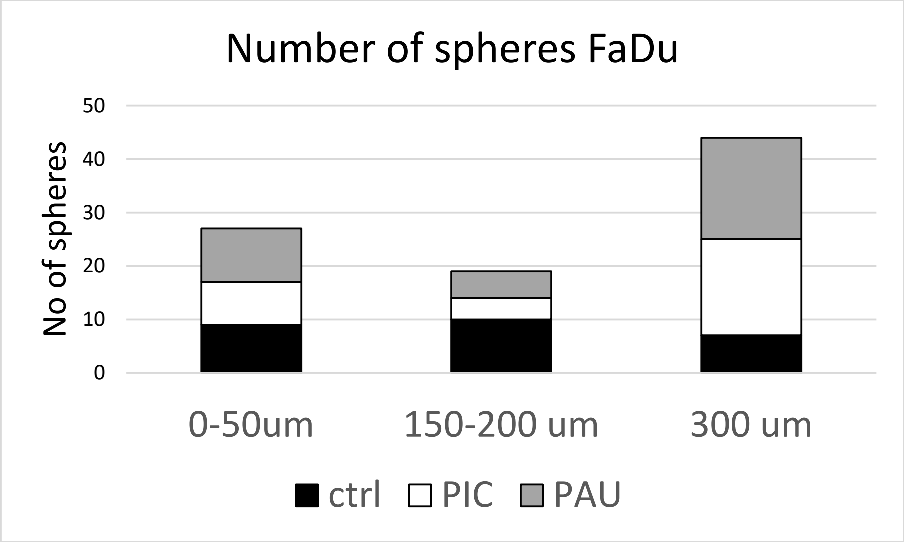

Figure S4. Number of spheres when FaDu cells were cultivated alone (ctrl) or with the addition of poly (I:C) (PIC) or poly (A:U) (PAU).

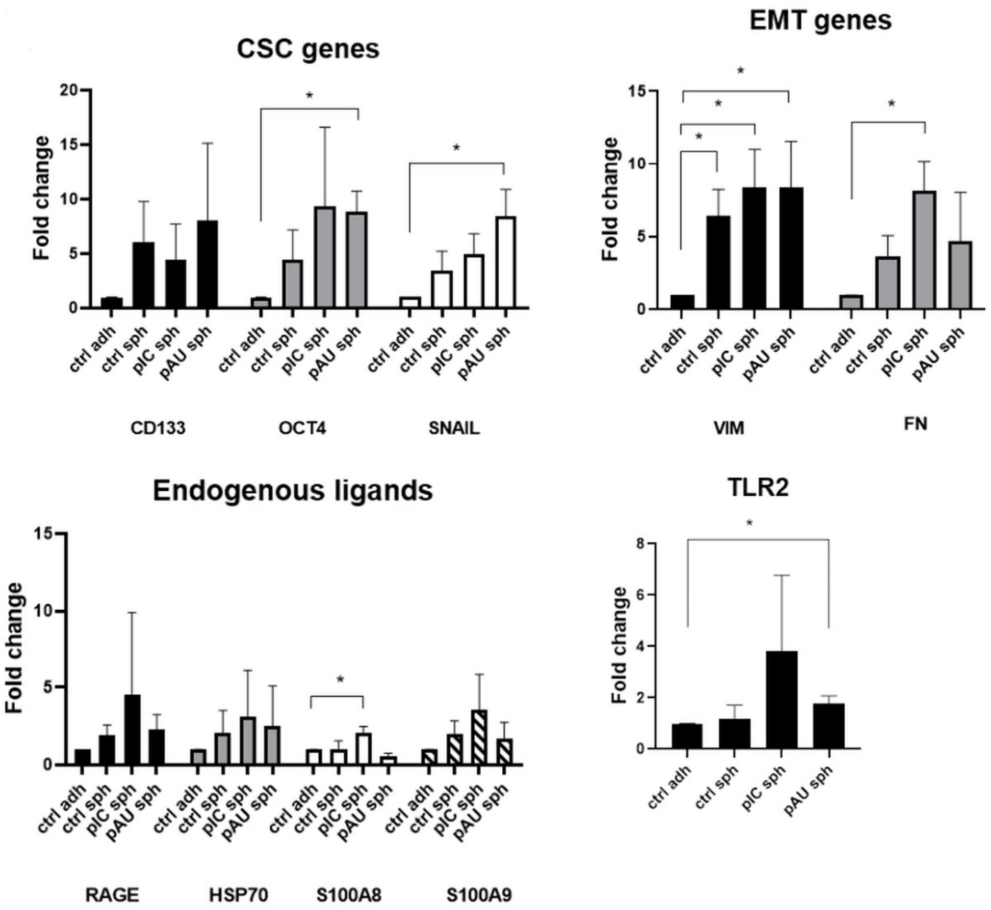

Figure S5. TLR3 induces the expression of damage-associated molecular patterns (DAMPs), CSC genes, EMT genes and TLR2 in FaDu cells

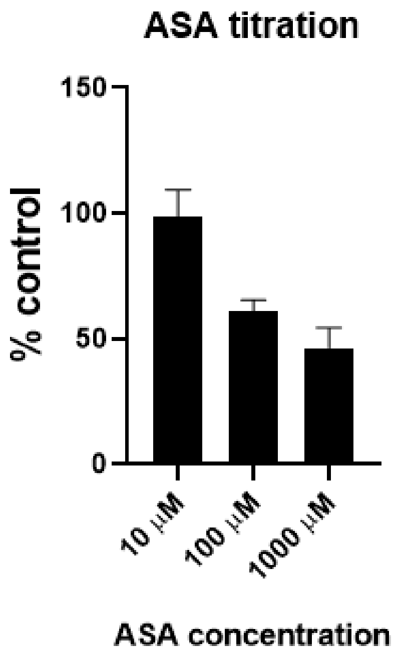

Figure S6. The titration of aspirin concentration by MTT assay after 48 hours

Original blots

S100A9

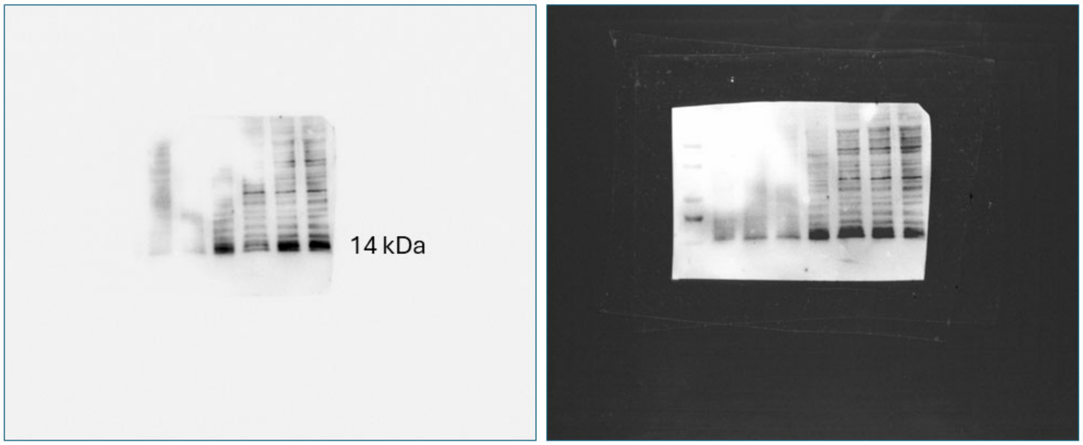

RAGE

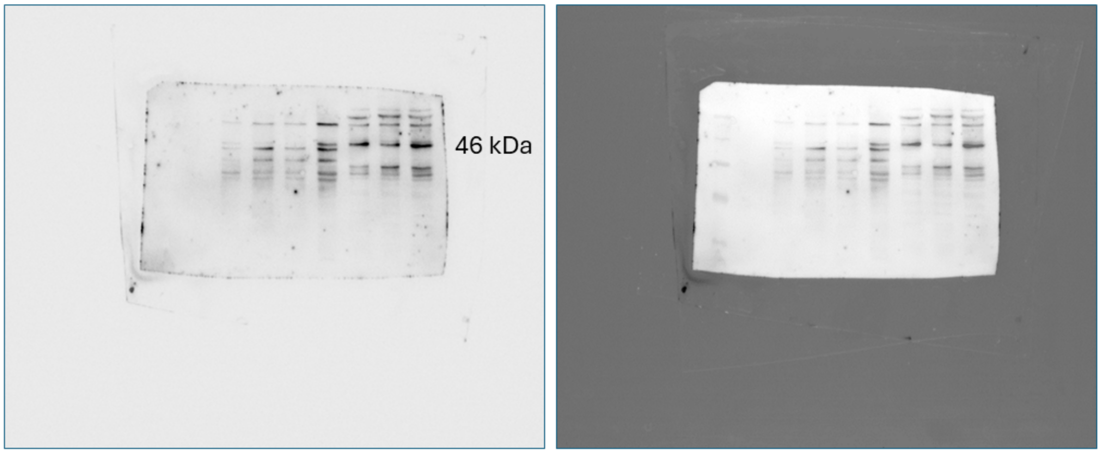

HMGB1

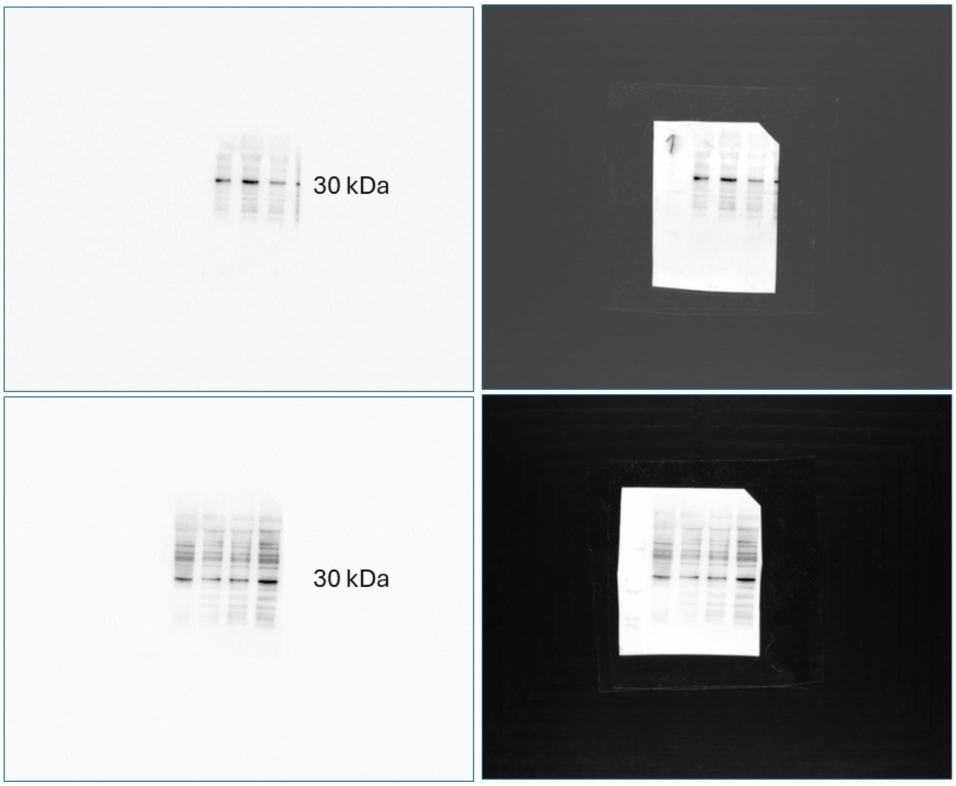

HSP70

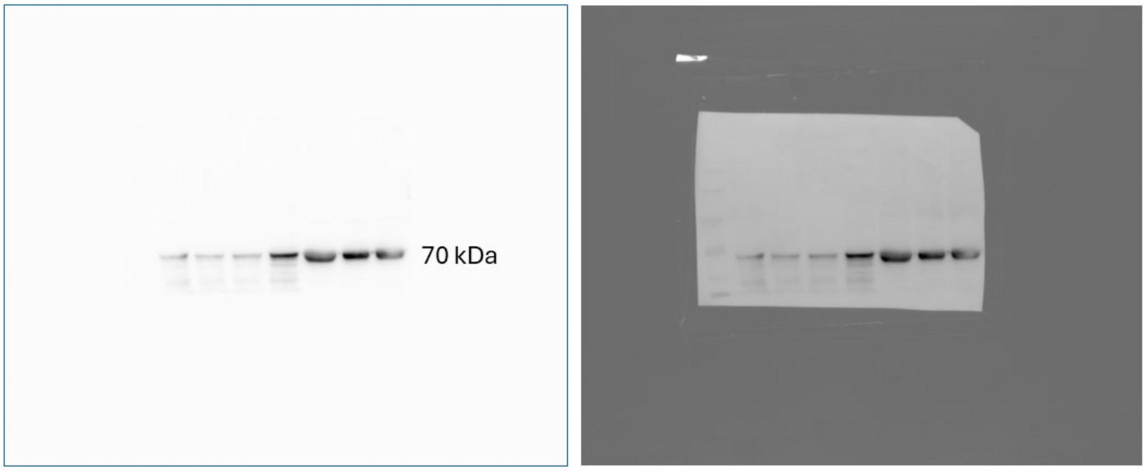

TLR4

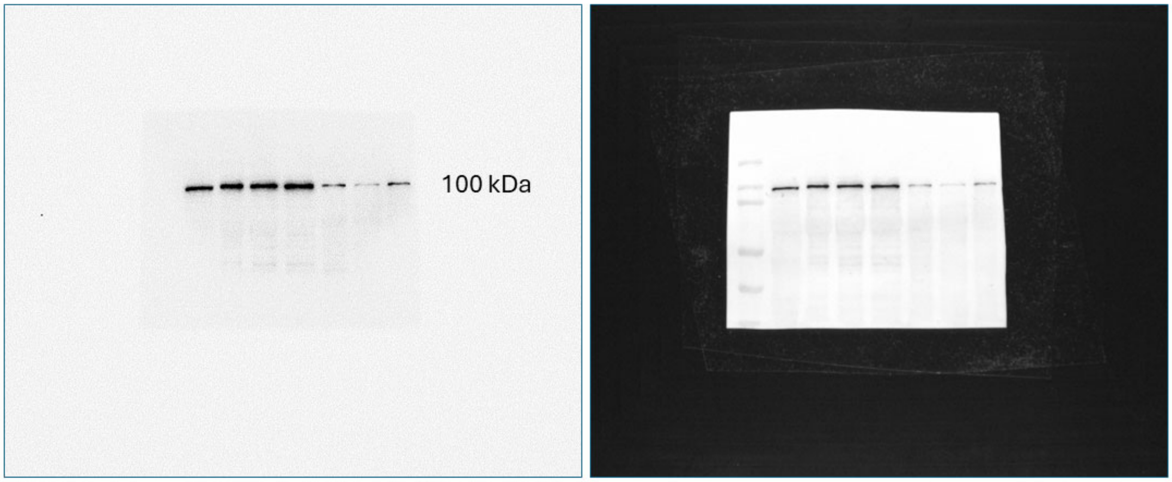

ALDH1A1

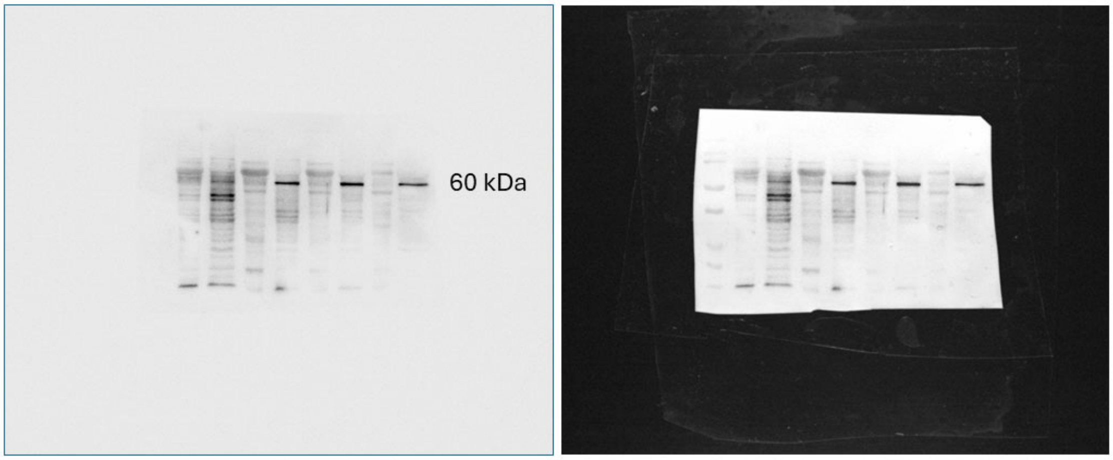

PARP
0 Gy

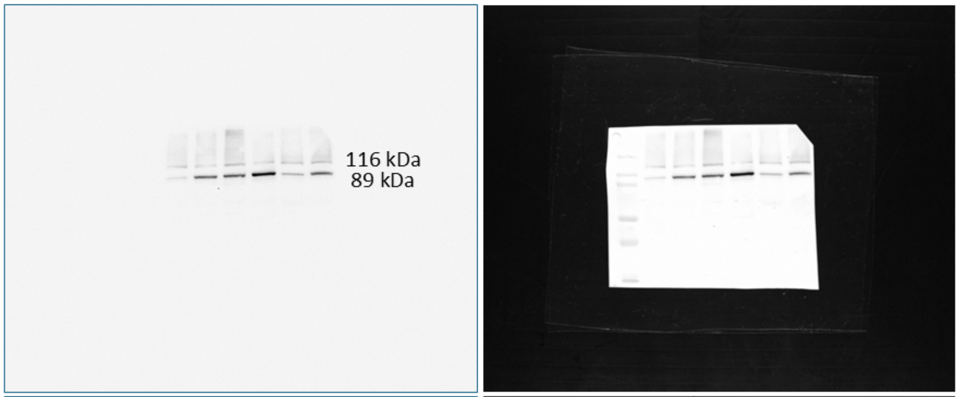

PARP
2 Gy

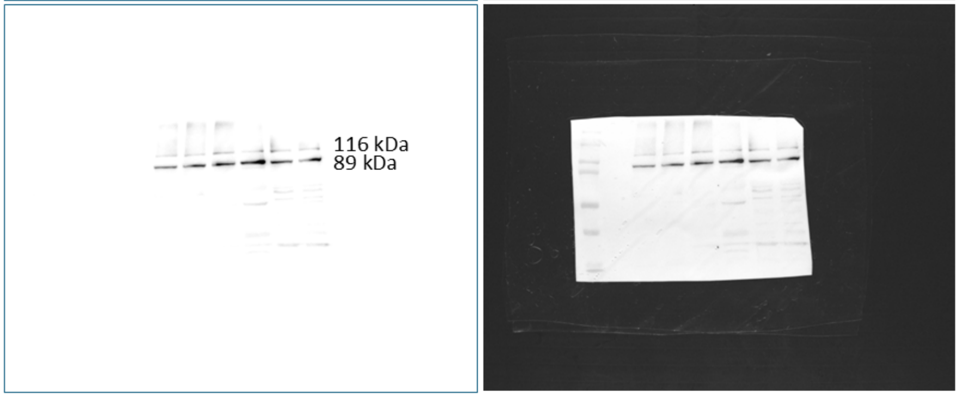

ABCG2
0 Gy

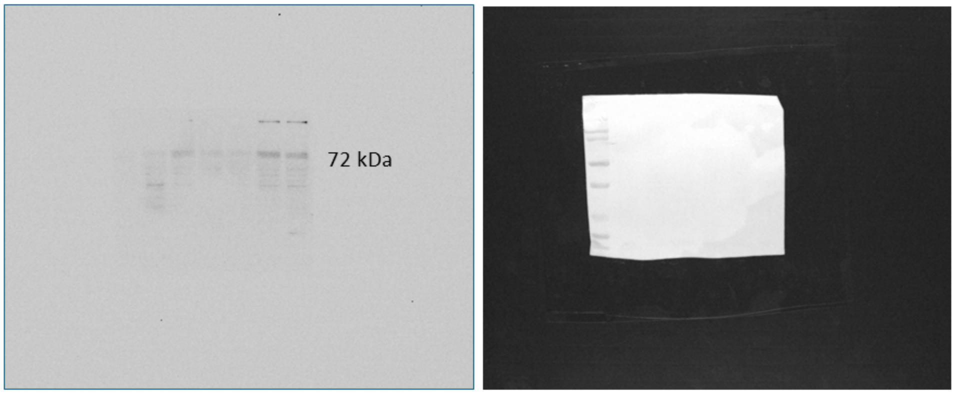

ABCG2
2 Gy

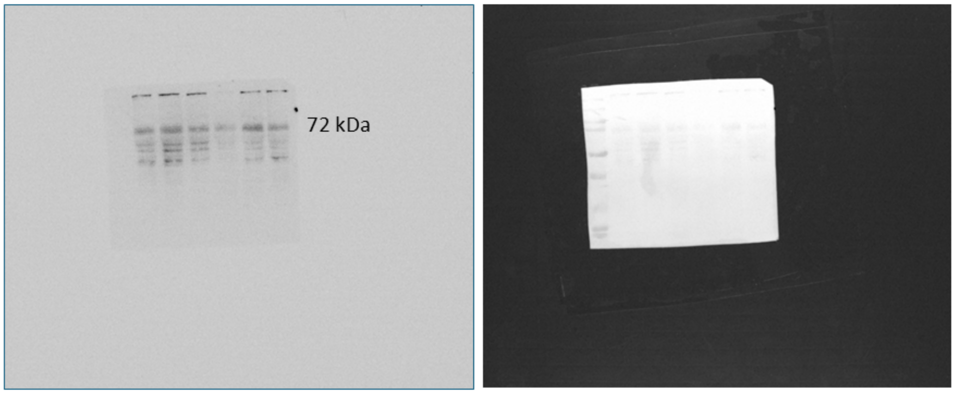

Supplement: Supplementary file 1 [file cancers-17-02157-s001.zip › cancers-3673730-supplementary.pdf]
